# Supplementary material for: The genome sequence of the Cistus Forester, Adscita geryon (Hübner, [1813]) (Lepidoptera: Zygaenidae)
Source: Wellcome Open Res. 2025 Oct 10;10:561. [Version 1] doi: 10.12688/wellcomeopenres.24954.1 (PMC12645097; doi:10.12688/wellcomeopenres.24954.1)
Supplement: Peer review history

# Reviewer report: Reviewer response for version 1

**DOI:** 10.21956/wellcomeopenres.27487.r137601

**Published:** 2025-12-27

**Authors:** Aleksandra Gwiazdowska

**Affiliations:** 1 Polish Academy of Sciences, Twarda, Poland

The authors described the genome of the Cistus Forester (Adscita geryon). The introduction contained information about the species. The photograph of the Cistus Forester in nature was fine, but I would like to see a photograph of the voucher that the authors used to do a genome or I would like to have information where I can find a photograph of the voucher. The methods are described in great detail, and each part has a link to see the details on the web side. The pipeline is also available online.

 In summary, the results and methods are available and reproducible. The reference genome will help for future studies.

Are sufficient details of methods and materials provided to allow replication by others?

Yes

Is the rationale for creating the dataset(s) clearly described?

Yes

Are the datasets clearly presented in a useable and accessible format?

Yes

Are the protocols appropriate and is the work technically sound?

Yes

Reviewer Expertise:

Population Genetics, Conservation Genetics

I confirm that I have read this submission and believe that I have an appropriate level of expertise to confirm that it is of an acceptable scientific standard.

# Reviewer report: Reviewer response for version 1

**DOI:** 10.21956/wellcomeopenres.27487.r138610

**Published:** 2025-11-24

**Authors:** Henk den Bakker

**Affiliations:** 1 University of Georgia, Griffin, Griffin, USA

This manuscript describes the sequencing of the genome of the Cistus Forester moth, Adscita geryon. It provides an introduction to the natural history of the species and a detailed description of the genome sequencing and associated bioinformatics protocols. 

 I have a few small suggested edits:

 1. Can the author address what the rationale was for sequencing  A. geryon, and not the more common species Adscita statices.

 2. In the last paragraph of the back ground section ...The branching pattern of Adscita,.. please change to ...The phylogeny of Adscita... 

 3. In the Nucleic acid extraction subsection of the Methods section, in the sentence before the last sentence of the second paragraph ...and a yield of 2 717.54 ng, with a fragment size of 16.4 kb. I assume it is an average fragment size. Please correct if that assumption is correct.

Are sufficient details of methods and materials provided to allow replication by others?

Yes

Is the rationale for creating the dataset(s) clearly described?

Yes

Are the datasets clearly presented in a useable and accessible format?

Yes

Are the protocols appropriate and is the work technically sound?

Yes

Reviewer Expertise:

Bioinformatics, phylogenetics, systematics

I confirm that I have read this submission and believe that I have an appropriate level of expertise to confirm that it is of an acceptable scientific standard.

# Reviewer report: Reviewer response for version 1

**DOI:** 10.21956/wellcomeopenres.27487.r138612

**Published:** 2025-11-24

**Authors:** Pritha Dey

**Affiliations:** 1 National Centre for Biological Sciences-TIFR, Bengaluru, India

The manuscript is overall well-written, concise. Here are a few comments:

 1) While the technical details are adequate, the manuscript will benefit from a brief understanding of why Adscita geryon is evolutionarily relevant-with details about habitat specificity and phylogenetic placement with the Zygaenidae family

 2) It would help the readers if the biological relevance of retaining two haplotypes of different lengths is highlighted. 

 Also, would subsequent genomic analysis use Haplotype 1 over Haplotype 2, if yes, why

Are sufficient details of methods and materials provided to allow replication by others?

Yes

Is the rationale for creating the dataset(s) clearly described?

Yes

Are the datasets clearly presented in a useable and accessible format?

Yes

Are the protocols appropriate and is the work technically sound?

Yes

Reviewer Expertise:

Taxonomy, evolutionary ecology

I confirm that I have read this submission and believe that I have an appropriate level of expertise to confirm that it is of an acceptable scientific standard.

# Reviewer report: Reviewer response for version 1

**DOI:** 10.21956/wellcomeopenres.27487.r135946

**Published:** 2025-11-06

**Authors:** Abdoallah Sharaf

**Affiliations:** 1 University of Konstanz, Konstanz, Germany

The article is scientifically sound in its current form, with no major flaws in rationale, methods, reproducibility, or data presentation. All evaluation questions can confidently be answered “Yes,” indicating high technical quality, methodological transparency, and data integrity.

 The work aligns with the standards and best practices of the *Darwin Tree of Life* (DToL) and *Earth BioGenome Project* initiatives, delivering a reference-quality genome for *Adscita geryon*. The assembly achieves excellent metrics (N50 = 24.94 Mb, BUSCO completeness = 98.0%), demonstrating careful experimental design, data handling, and quality control.

 No substantive revisions are required for scientific soundness. The article represents a valuable and well-executed contribution to biodiversity genomics and Lepidopteran research.

Are sufficient details of methods and materials provided to allow replication by others?

Yes

Is the rationale for creating the dataset(s) clearly described?

Yes

Are the datasets clearly presented in a useable and accessible format?

Yes

Are the protocols appropriate and is the work technically sound?

Yes

Reviewer Expertise:

genomics, bioinformatics, and biodiversity research

I confirm that I have read this submission and believe that I have an appropriate level of expertise to confirm that it is of an acceptable scientific standard.
